# Impact of maternal antibodies and infant gut microbiota on the immunogenicity of rotavirus vaccines in African, Indian and European infants: protocol for a prospective cohort study

**DOI:** 10.1136/bmjopen-2017-016577

**Published:** 2017-03-29

**Authors:** Kuladaipalayam Natarajan C Sindhu, Nigel Cunliffe, Matthew Peak, Mark Turner, Alistair Darby, Nicholas Grassly, Melita Gordon, Queen Dube, Sudhir Babji, Ira Praharaj, Valsan Verghese, Miren Iturriza-Gómara, Gagandeep Kang

**Affiliations:** 1 Division of Gastrointestinal Sciences, Christian Medical College, Vellore, Tamil Nadu, India; 2 University of Liverpool, Liverpool, UK; 3 Alder Hey Children's NHS Foundation Trust, Liverpool, UK; 4 London School of Hygiene and Tropical Medicine, London, UK; 5 Malawi College of Medicine, Malawi; 6 Department of Child Health, Christian Medical College, Vellore, Tamil Nadu, India

**Keywords:** Rotavirus vaccine, Immunogenicity, Microbiota, Maternal antibodies, Polio vaccine

## Abstract

**Introduction:**

Gastroenteritis is the leading cause of morbidity and mortality among young children living in resource-poor settings, majority of which is attributed to rotavirus. Rotavirus vaccination can therefore have a significant impact on infant mortality. However, rotavirus vaccine efficacy in Sub-Saharan Africa and Southeast Asia is significantly lower than in high-income countries. Maternally derived antibodies, infant gut microbiota and concomitant oral polio vaccination have been proposed as potential reasons for poor vaccine performance in low-income settings. The overall aim of this study is to compare the role of maternally derived antibodies and infant gut microbiota in determining immune response to rotavirus vaccine in high-income and low-income settings, using the same vaccine and a similar study protocol.

**Methods and analysis:**

The study is an observational cohort in three countries—Malawi, India and UK. Mothers will be enrolled in third trimester of pregnancy and followed up, along with infants after delivery, until the infant completes two doses of oral rotavirus vaccine (along with routine immunisation). The levels of prevaccination maternally derived rotavirus-specific antibodies (IgG) will be correlated with infant seroconversion and antibody titres, 4 weeks after the second dose of rotavirus vaccine. Both within-country and between-country comparisons of gut microbiome will be carried out between children who seroconvert and those who do not. The impact of oral polio vaccine coadministration on rotavirus vaccine response will be studied in Indian infants.

**Ethics and dissemination:**

Ethical approvals have been obtained from Integrated Research Application System (IRAS, NHS ethics) in UK, College of Medicine Research and Ethics Committee (COMREC) in Malawi and Institutional Review Board (IRB), Christian Medical College, Vellore in India. Participant recruitment and follow-up is ongoing at all three sites. Analysis of data, followed by publication of the results, is expected in 2018.

**Strengths and limitations of this study**

- This study will for the first time provide data across populations on the impact of maternally derived immunity and intestinal microbiota on rotavirus vaccine take and immunogenicity in the infant.
- This study will evaluate the performance of the rotavirus vaccine in the routine schedule in two developing countries where oral polio vaccine will be soon phased out to be replaced with inactivated polio vaccine as per the Global Polio Eradication Initiative Strategic Plan for 2013–2018.
- Limitations include different ages at immunisation between the UK (8 and 12 weeks of age) and Malawi and India (6 and 10 weeks of age) because of differences in national vaccination schedules.
- Potential variations in breastfeeding practices are also a limitation.
- Measurement outcomes may not correlate with clinical outcomes (IgA may not be a correlate of protection).

## Introduction

This protocol followed published guidelines for protocols for observational studies, using Strengthening the Reporting of Observational Studies in Epidemiology (STROBE) checklist for cohort studies.^1^

### Background

Gastroenteritis is the leading cause of morbidity and mortality worldwide and is the second commonest cause of death among young children living in resource-poor settings.^2^ The year 2013 witnessed over 558 000 deaths due to diarrhoea among children in the under-5 age bracket.^3^ Rotavirus (RV) is the predominant aetiological agent of moderate-to-severe gastroenteritis in infants living in resource-poor settings.^4^ Diarrhoeal diseases attributed to RV constitute a major proportion of illness and death among children <5 years, especially in low-income countries.^5^ ^6^ Oral vaccines provide an effective as well as a pragmatic approach to alleviate the burden of enteric diseases for which vaccines are available. RV vaccination has the potential for significant reductions in infant mortality due to diarrhoea.^7–9^

Enteric vaccines must ideally elicit a robust local immune response. While live attenuated vaccines such as the oral polio vaccine (OPV) have had success in eliminating the circulation of poliovirus from the majority of countries, there is ample evidence that oral vaccines against poliomyelitis and cholera are less immunogenic among children living in the low-income countries, and therefore their effectiveness is compromised in those populations which need them most.^10^ RV vaccine efficacy in Sub-Saharan African countries and in Southeast Asia is significantly lower than in high-income countries.^11–13^ Maternally derived antibodies have been proposed as one potential reason for poor RV vaccine efficacy in populations in low-income settings with high exposure to RV.^14^ ^15^ High titres of RV-specific secretory IgA antibodies in breast milk influence the vaccine strain immunogenicity even though withholding breast feeding for a short duration does not influence the measured immune response to vaccines.^16^ Transplacental transfer of malaria-specific IgG antibodies to the infant is influenced by the maternal HIV status and a similar phenomenon may be plausible for other antibodies in countires.^17^ This is important because in countries where the RV burden is high and early exposure to RV is common, maternally derived immunity may be critical in protecting newborn infants from RV disease prior to immunisation. A low maternal exposure to RV in developed countries may result in lower levels of passive transfer to the infant. Hence, the extent to which pre-existing RV-specific IgG affects vaccine immunogenicity in infants and their patterns in different countries need to be studied.

The human gut microbiota has a role in controlling metabolic and physiological functions like nutrient usage, synthesis of vitamins, intestinal and immunological design, development and maturation.^18–21^ Gnotobiotic animal models provide insights into the mechanisms by which the intestinal microbiota shape the immune system and responses.^22^ Studies in Malawi and Bangladesh have shown strong associations between the gut microbiome composition and malnutrition.^23^ ^24^ Intestinal microbiota will likely influence the immune responses to vaccines. A role in influenza vaccination has been recently reported.^25^ The gut microbiota has been shown to influence enteric virus replication in mice.^26^ Oral enteric live attenuated vaccines require replication in the intestinal tract and hence the gut microbiota may directly and/or indirectly influence vaccine strain replication, thus affecting the immune response. Studies have also highlighted the role of breast milk microbiota in shaping the infant gut microbiome composition thereby influencing immune system maturation.^27^ ^28^ Understanding the role of the microbiota in vaccine responses, in different populations, is essential to understand what determines the ability of vaccine strains to replicate in the gut and their interactions with the local environment.

### Rationale for the current study

This project will investigate the key maternal and infant factors that may reduce RV vaccine performance in infants living in resource-poor settings. RV is the leading cause of severe gastroenteritis in infants worldwide where the vaccine is not in routine use. Almost half a million children die of RV gastroenteritis each year, mainly in the developing world. Live, attenuated, orally administered RV vaccines have the potential to greatly reduce global diarrhoea deaths but for unexplained reasons they work less well in the lowest income, highest disease burden countries where they are needed most. Thus, in Sub-Saharan Africa and Southeast Asia, vaccine efficacy against severe RV diarrhoea ranges between 43% and 66% compared with >90% in high-income countries.

Although levels of maternal immunity to RV are known to be higher in mothers living in low-income countries, their impact at the time of immunisation on RV vaccine take and immunogenicity in the infant has not been investigated. Also, the relationship between systemic antibodies and secretory antibodies in breast milk is not clear; although studies in which breast feeding has been withheld immediately prior to RV immunisation have not had any significant impact on the vaccine immunogenicity. However, these studies do not rule out the interference of breast milk antibodies on vaccine take.

Despite efforts to study the interface between commensal bacteria and host immune responses, the role of the intestinal microbiota on vaccine efficacy remains underinvestigated, and the impact of the composition of microbial communities in the gut on live oral vaccine efficacy has not been elucidated to date. All of these underline the fact that this study will yield information that is required to understand the performance of RV vaccines in developing countries and thus develop strategies to improve them.

### Aim and objectives

**Figure 1:**
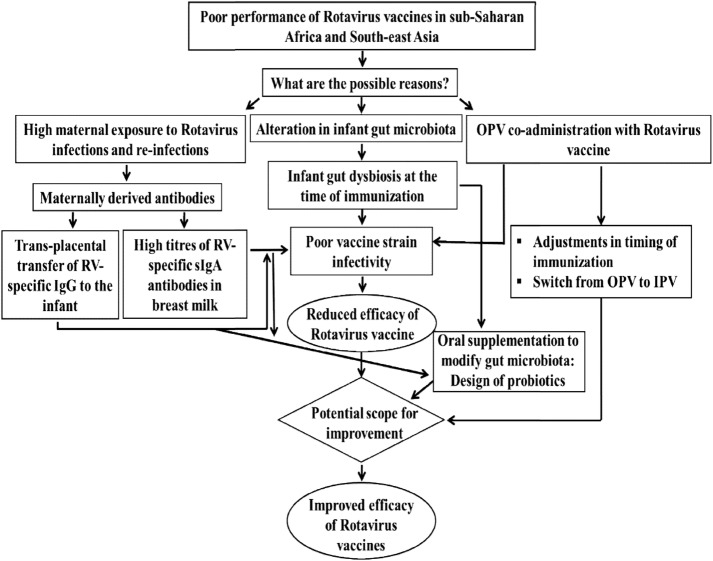
Conceptual framework of the rotavirus vaccine immunogenicity study.

The overall aim of this study is to investigate the role of maternally derived antibodies and the infant gut microbiome in determining the IgA immune response to RV vaccine in high-income and low-income countries by comparison of three populations using the same vaccine and a similar study protocol (figure 1).

*Objective 1*: To investigate the role of maternally derived antibodies in determining RV vaccine take and immunogenicity in infants.

#### Primary objective

To correlate levels of prevaccination maternally derived RV-specific antibodies (IgG) with infant seroconversion and antibody titres (immunogenicity) 4 weeks after two doses of Rotarix administered at 6/8 and 10/12 weeks of age. In addition, RV vaccine shedding will be assessed in stool at day 7 after each vaccine dose as a proxy for vaccine take.

#### Secondary objectives

- To evaluate the frequency and impact of early exposure to RV infection prior to vaccination on RV vaccine take and immunogenicity.
- To correlate RV-specific IgA in breast milk with infant response to vaccine and levels of maternal serum IgG and IgA with breast milk IgA.

*Objective 2*: To investigate if dysbiosis at the time of vaccination is associated with poor RV vaccine take and low immunogenicity.

#### Primary objective

To compare gut microbial communities among infants who seroconvert with those who do not, to determine whether infants who fail to seroconvert following RV vaccination have a distinct microbiota.

#### Secondary objectives

- Role of dysbiosis in intestinal inflammatory disease: To determine whether infants with biomarkers indicative of intestinal inflammation, as measured by faecal α-1 antitrypsin (AAT), and myeloperoxidase (MPO) levels, and of systemic inflammation, measured by levels of acid glycoprotein in blood, are associated with an altered intestinal microbiota.
- To study the role of maternal microbiota imprinting on the development of infant dysbiosis by studying how the gut microbiota of each infant is determined by the maternal gut and breast milk microbiota and how it evolves from shortly after birth to the time of vaccination.

*Objective 3*: To investigate the role of concomitant administration of OPV in RV vaccine take and immunogenicity.

The impact of OPV coadministration on RV vaccine response will be investigated in India by including an additional arm where OPV will be replaced by inactivated polio vaccine (IPV) at weeks 6 and 10 (following OPV at birth). This will allow comparisons in children with coadministered OPV and IPV. This will provide crucial data that may enable prediction of the performance of RV vaccines when countries switch over from OPV to IPV.

## Methods and analysis

### Study design and setting

The study is an observational cohort at all the three sites. The study will recruit pregnant women in their third trimester and subsequently their newborn infants (mother–infant pairs) from the Zingwangwa Health Centre in Blantyre, Malawi; the Chinnallapuram Community Clinic in Vellore, India, and the Liverpool Women's Hospital in Liverpool, UK.

### Inclusion criteria

- pregnant women consenting to participate in the study;
- women willing to stay in the study area for 4 months following delivery.

### Exclusion criteria

- congenital immune deficiency;
- chronic renal or liver failure;
- other chronic illnesses which may affect the immune response;
- mothers with non-singleton pregnancy;
- infant born with low birth weight/preterm (<34 weeks gestation), congenital anomalies and other neonatal complications requiring prolonged hospitalisation.

### Malawi study site description

Blantyre is (15.45°N 35.00°E) located in the southern region of Malawi and is the second largest city in the country.^29^ In 2008, the crude birth rate in Blantyre was 33.5 per 1000, with a fertility rate of 5.4 per woman, and infant (<1 year of age) and child (1–4 years of age) mortality rates being 98 and 55 per 1000 live births, respectively, and a fertility rate of 5.^30^ The city is served by 11 health centres and one tertiary referral government hospital, The Queen Elizabeth Central Hospital that provides free healthcare to the population. Recruitment for the study will take place at the Zingwangwa Health Centre, located in the Zingwangwa urban slum. The health centre will provide antenatal and delivery services, primary care, HIV and immunisation clinic services and vaccination by research clinician in liaison with the government nursing and midwifery staff. The community follow-up of the mother–infant pairs will be done by study field workers.

### Vellore study site description

Vellore (12.92°N 79.13°E) is a town in Tamil Nadu, the southernmost state of India. The site will enrol mother–infant pairs from the areas of Ramnaickapalayam, Chinnallapuram, Kaspa, Vasanthapuram and Vellore old town. Ramnaickapalayam, Chinnallapuram, Kaspa and Vasanthapuram are semiurban slums situated in the western outskirts of Vellore with a population of about 49 000 and a birth rate of 17.5 per 1000 whereas Vellore old town is an urban slum with a population of 22 000 and a birth rate14.6 of per 1000 population. ‘Beedi-work’ (indigenous cigarette made of unprocessed tobacco, manually wrapped in ‘tendu’*—Diospyros melanoxylon* leaf) is the predominant occupation, followed by unskilled labour. The majority of the population is dependent on income through daily wage-based jobs. Government urban health centres in the area provide free healthcare to the residents within the study area, and a government teaching hospital is located ∼5 km away. The Christian Medical College (CMC), Vellore, a not-for-profit organisation and its two outreach units—the Community Health and Development (CHAD) and the Low Cost-effective Care Unit (LCECU)—are located within a few kilometres of the study area. As per the sample registration survey conducted by the Government of India, the birth and infant mortality rates for urban Tamil Nadu for the year 2013 are 15.8 live births per 1000 population and 22 deaths per 1000 live births, respectively.^31^ A service clinic (the Chinnallapuram Community Clinic) provides basic healthcare to all children under the age of 5 years. Those children requiring hospitalisation or specialised care are referred to the secondary (CHAD) or tertiary care hospital (CMC), as deemed necessary by the attending physicians. The study will be conducted in the research wing of the clinic. Trained field staff and study nurses who have established a rapport with the community will be involved in enrolment, immunisation and follow-up of the mother–infant pairs.

### Liverpool study site description

Liverpool is a city situated in the North West of England, with an estimated population of 478 600 inhabitants. Infant mortality rate in the city, 25 recorded in 2013, is above regional and national levels, but is not statistically significant. Fertility rates in Liverpool are below national levels. The percentage of women in Liverpool initiating breast feeding within the first 48 hours of birth was 53.2% in 2013–2014, the lowest among the main English cities, and significantly below the regional and national levels. Immunisation coverage was 94.5% in 2013–2014.^32^

Participants will be recruited at the Liverpool Women's Hospital (LWH). The LWH provides free maternity services for the Liverpool city region encompassing the local authority districts of Halton, Knowsley, Liverpool, Sefton, St Helens and Wirral with a total population of 1 506 935 of predominantly white, British or Irish heritage (93.2%). Approximately 8000 babies are delivered annually at the LWH.

### Enrolment and follow-up of mother–infant pairs

**Figure 2:**
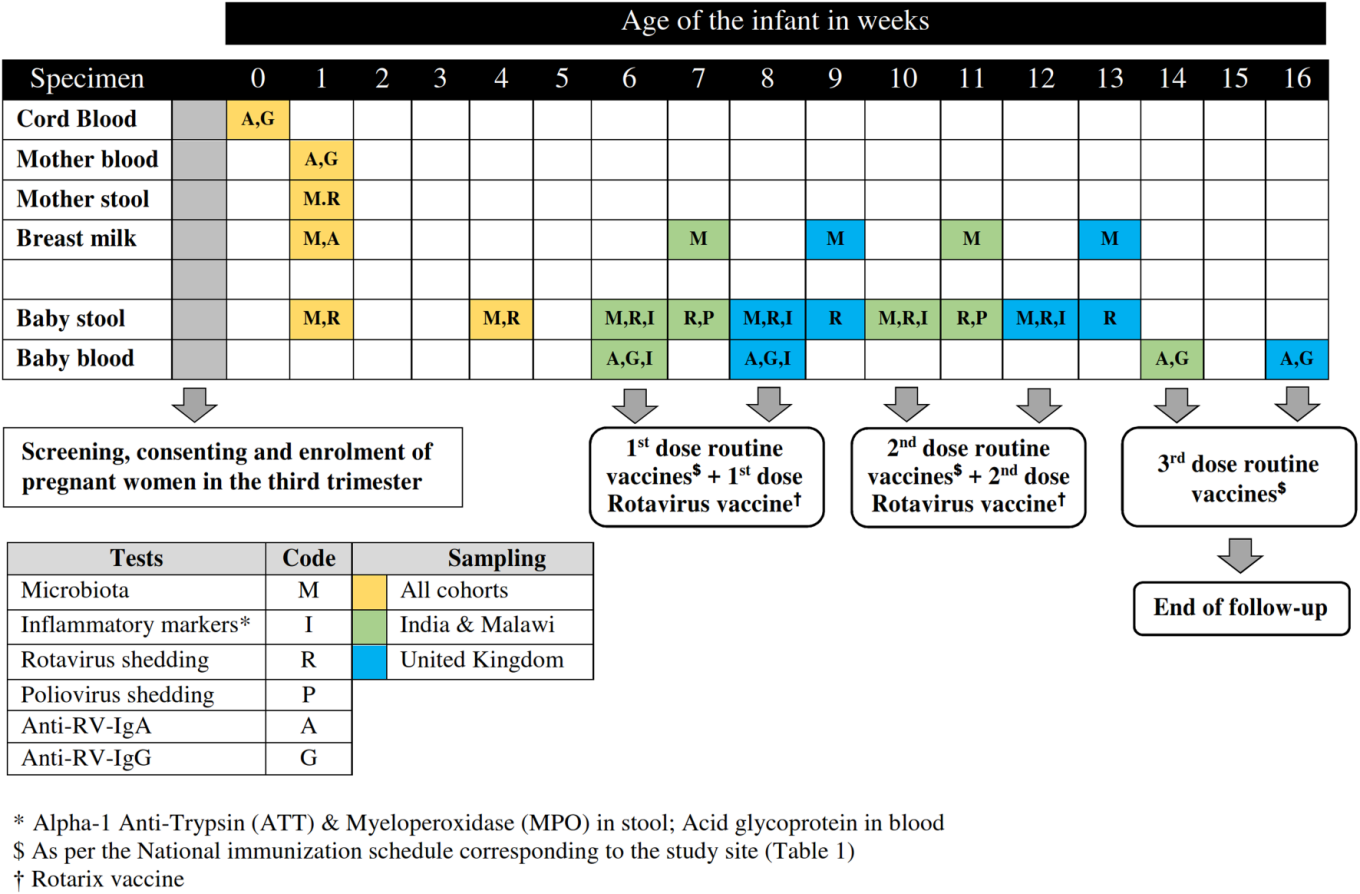
Schematic representation of enrolment and follow-up of the mother–infant pairs at Malawi, India and UK.

Pregnant women in their third trimester available for follow-up for at least 4 months postnatally will be informed about the study by the visiting field staff or by post and be invited to participate in the study. Women with a prior diagnosis of congenital immune deficiency, chronic renal or liver failure, other chronic illnesses which might affect immune response (other than HIV) and mothers with non-singleton pregnancy will be excluded. Following the initial screening, women who subsequently deliver preterm babies (<34 weeks of gestation) will be excluded. After screening and informed consent is obtained, body mass index, haemoglobin and HIV status will be documented. A blood sample of 5 mL will be collected from the mother by the phlebotomist for baseline anti-RV IgG and IgA levels. Following delivery, mode and place of delivery, gender and birth weight will be documented by the staff conducting the delivery; sample collection, as outlined in figure 2, will be conducted by the field staff/phlebotomist/study nurse as per the calendar generated immediately following the delivery of the infant. The mother–infant pair will be followed until the infant completes the third dose of routine immunisation, which marks the end of the study. Except the birth dose of BCG, Hepatitis B and OPV (India) which are usually administered at the point of delivery, all routine immunisations along with two doses of RV vaccine (Rotarix) will be administered to the infants as per the site's national immunisation schedule (table 1). History of prelacteal feeds and initiation of breast feeding details will be collected. At every sampling point, details of breast feeding, prior and current illness if any and antibiotic usage will be documented. The levels of prevaccination maternally derived RV-specific antibodies (IgG) will be correlated with infant seroconversion and antibody titres (immunogenicity) 4 weeks after two doses of RV vaccine are administered.

**Table 1:** National immunisation schedules of the three study sites between birth and 4th month

	Birth	6th week	8th week	10th week	12th week	14th week	16th week
Malawi	BCG and OPV-0	OPV-1, DPT-HepB-Hib-1, PCV-1 and RV-1		OPV-2, DPT-HepB-Hib-2, PCV-2 and RV-2		OPV-3, DPT-HepB-Hib-3, PCV-3 and RV-3	
India*	BCG, OPV-0 and Hep B-0	OPV-1, DPT-HepB-Hib-1		OPV-2, DPT-HepB-Hib-2		OPV-3, DPT-HepB-Hib-3	
UK			IPV-1, DPT-HepB-Hib-1, PCV-1, MenB and RV-1		IPV-2, DPT-HepB-Hib-2, PCV-2, MenC and RV-2		IPV-3, DPT-HepB-Hib-3, MenB and PCV-3

*RV is an optional vaccine in India (Rotarix administered as two doses in the 6th and 10th week).

DPT, diphtheria pertussis tetanus; Hep B, hepatitis B; Hib, haemophilus influenza type b; IPV, inactivated polio vaccine; MenB, meningococcal B; MenC, meningococcal C; OPV, oral polio vaccine; PCV, pneumococcal vaccine; RV, rotavirus vaccine.

### Sample size

Approximately 40% of the Indian and African infants are expected to seroconvert following two doses of RV vaccine.^33–35^ A sample size of 150 infants would thereby give a power of 80% to detect a twofold higher mean concentration of RV-specific IgG antibodies at 6 weeks in those who fail to seroconvert compared with those who seroconvert, assuming an SE of 1 log_e_ International Unit, and 90% power to detect a correlation between the concentration of RV-specific serum IgA at 6 and 14 weeks.^7^ In the UK, 95% of infants are expected to seroconvert. The levels of RV-specific serum IgA antibody titres of infants in the UK will be compared with those in India and Malawi. Hence, a sample size of 50 in the UK would give a power of 79% to compare antibody concentrations according to seroconversion status across the study sites with a 10% allowance for potential loss to follow-up, giving a sample size 165 mother–infant pairs in India and Malawi, and 55 mother–infant pairs in the UK.

The sample size calculated is also powered to >95% to detect a significant difference in the (Shannon) diversity of the gut microbiota at the time of vaccination between infants who seroconvert and those who do not, both within the infants at India and Malawi and in comparison with those at the UK, based on in-house observations of this variance in 6-week-old infants (unpublished data) and a difference of 0.5 consistent with that observed during episodes of malnutrition or diarrhoea.^23^ It is expected that ∼21 and 8 infants would get infected or develop RV diarrhoea, respectively, before the 6th week immunisation visit in Vellore, and similarly in Malawi (data not available for UK).^11^ ^33^ Maternally derived antibody and microbiota will be compared in the infants who had RV diarrhoea with the other infants in the study, but substantial power in these analyses may not be possible.

To determine the potential interference of OPV coadministration with RV vaccine apropos to the differences in immunogenicity between the UK and Indian infants (where bivalent OPV continues to be a part of the national immunisation schedule), OPV will be replaced with IPV for an additional cohort of 90 infants recruited in India that is powered to 83% to detect a 20% difference in seroconversion following two doses of RV vaccine, assuming a 10% loss to follow-up.^36^

### Outcome measures

The levels of prevaccination maternally derived RV-specific antibodies (IgG) will be correlated with infant seroconversion and antibody titres (immunogenicity) 4 weeks after two doses of RV vaccine are administered at 6th and 10th week of age (WHO-recommended schedule applied in India and Malawi) or 8th and 12th week of age (UK national recommendation) (table 1). In addition, RV shedding in stool on day 7 following each vaccine dose will be tested as an additional marker of vaccine take (figure 2).

Comparison of gut microbial communities will be performed among infants who seroconvert with those who do not, both within and between populations, in order to determine whether infants who fail to seroconvert following RV vaccination have a distinct microbiota.

The impact of OPV coadministration on RV vaccine response in India will be studied by including an additional arm where OPV will be replaced by IPV at 6th and 10th week (following OPV at birth), to allow comparisons with Indian children coadministered OPV and with children in the UK who receive IPV. This will provide data that may enable prediction of the performance of RV vaccines when IPV is introduced in developing countries later, especially since a single dose of IPV in immunisation schedules of six states from November 2016 in India.^37^ ^38^

### Laboratory methods

#### Specimen processing and storage

All specimens will be transported to the receiving laboratory at each site within 24 hours of collection. Blood samples will be separated for plasma and be frozen until further testing. Stool and breast milk specimens will be stored frozen at −70°C.

#### Immunology

RV-specific IgG or IgA antibodies will be detected using standardised quantitative ELISA methods that have been validated and are certified for regulatory submissions for RV vaccine trials. Levels of RV-specific IgG and IgA will be measured in the mother's blood, cord blood and infant's blood specimens to allow for the assessment of transplacentally acquired antibodies and possibly the duration of maternally transferred protection in the infant (IgG) at the following time points: early exposure (birth to the first dose of RV vaccine), prior to RV vaccine immunisation, potential RV infections (infant IgA prior to vaccine dose) and seroconversion post-RV immunisation (infant IgA at 14th or 16th week) (table 2).

**Table 2:** Summary of the laboratory tests and assays

Domain	Laboratory parameter		Type of specimen	Method/assay
Immunology	Rotavirus (RV)-specific IgG antibodies		Mother's blood Cord blood Infant's blood	ELISA
	RV-specific IgA antibodies			
	Innate immunity activation markers		Infant's blood	TruCulture blood collection and whole-blood culture systems
Virology	Rotavirus shedding		Mother's stool Infant's stool	qPCR
	Poliovirus shedding (type 1 and 3)		Mother's stool Infant's stool	qPCR
Markers of inflammation	Local (gut) inflammation	α-1-Antitrypsin (ATT) Myeloperoxidase	Infant's stool	ELISA
	Systemic inflammation	Acid glycoprotein	Infant's blood	ELISA
Microbiota	Characterisation of microbiota composition		Mother's stool Breast milk Infant's stool	DNA extraction Amplification Next generation Sequencing

qPCR, quantitative PCR.

Innate immunity activation markers of enteric infection that could potentially affect the immune response to RV vaccine will be assessed in a subset of samples from Malawi and India where blood (1 mL) will be collected in a TruCulture whole blood culture tube with and without stimulant (0.5 mL in each), incubated and frozen for analysis of cytokine expression.^39^ Poly I:C (stimulant), which interacts with toll-like receptor 3, expressed in the membrane of B-cells, macrophages and dendritic cells, a synthetic analogue of dsRNA, which in turn is a molecular pattern associated with viral infection will enable the study of patterns of innate immune responses triggered by viral infections between children who seroconvert and those who do not following RV immunisation (table 2).

#### Virology

RV and poliovirus vaccine strains (1 and 3) will be detected in stool samples using previously validated Real Time/quantitative PCR assays (qPCR). A 10–20% stool suspension will be prepared in phosphate buffer solution prior to total nucleic acid extraction. The extracted nucleic acid will be used to generate cDNA by reverse transcription using random hexamers and this will serve as a template in the RV and OPV qPCRs.

RV vaccine take will be determined through detection of RV vaccine strain shedding in stool 1 week postvaccination, using Rotarix-specific qPCR. OPV shedding will be measured by detecting Sabin poliovirus types 1 and 3 shedding in stool, 1 week postvaccination, also by qPCR (table 2).

#### Markers of local (gut) inflammation

This will be assessed using commercially available ELISA for the detection of AAT and MPO in stool samples. Systemic inflammation will be assessed using a commercially available ELISA for the detection of acid glycoprotein in serum/plasma. These assays will be performed in each of the three laboratories using the same methods and the same batch of kits (table 2).

#### Microbiota

Samples for microbiota analysis will be stored for a maximum of 2 weeks prior to DNA extraction. Nucleic acid extraction will be carried out at each of the sites. Samples will be treated by bead beating and lysozyme prior to DNA extraction with the QiagenQIAamp DNA stool extraction kit (table 2). Blank water controls will be included in each extraction batch and run through as sequencing controls. Extracted DNA will be aliquoted and stored at −20°C prior to amplification. All sites will adopt the same SOPs to ensure that sample collection and DNA extraction and sequencing are standardised and that the data are comparable between sites.

All frozen DNA samples will be shipped to Liverpool, UK, undergo quality control and library construction using a Nextera v2 Dual Index Barcoding approach, before sequencing on the IIlumina HiSeq 2500 Rapid Run mode or alternatively the Illumina MiSeq platform. Approximately 10% of the samples will additionally be sequenced at Imperial College London to test the reproducibility and robustness of the protocols and allow comparison and validation.

The 16S rDNA PCR strategy will use a nested dual index protocol to amplify and barcode the variable V3–V4 region (319f—5′ ACTCCTACGGGAGGCAGCAG 3′ & 806r—5′ GGACTACHVGGGTWTCTAAT 3′) resulting in ∼469 base pair PCR product. Then barcoded 16s PCR products will be multiplexed and sequenced on the Illumina HiSeq 2500 or MiSeq platform to produce 2×250 base pair reads.

### Statistical analysis

#### Role of pre-existing IgG and RV exposure

The mean log-concentration of RV-specific antibody (IgG, IgA) measured at 6 weeks in infants who seroconvert or not following RV vaccination will be compared using Student's t-test or a non-parametric test as appropriate. The associations between seroconversion and maternal factors, including infection status and early infant exposure to RV infection, will be assessed using Student's t-test or χ^2^ test as appropriate and multivariate logistic regression will be performed to deduce interactions between these variables. Similar analyses will be performed for infants grouped according to the detection of RV shedding in the week following vaccination as a marker of vaccine take.

#### Microbiota

V3/V4 sequences will undergo a validated error correction protocol. Quality filtering, including the trimming of low-quality bases at the start and end of each read with Sickle (https://github.com/najoshi/sickle) and error correction using Bayes Hammer, will be performed prior to the assembly of paired reads with PEAR (V.0.9.1) or equivalent.^40^ ^41^ A size selection filter set to the expected amplicon size and BLAST searches ensure that PhiX spike in control is detected and removed.

These corrected and assembled reads will then be analysed with QIIME (Quantitative Insights Into Microbial Ecology).^42^ USEARCH will be run using de novo and open reference operational taxonomic units (OTU) clustering methods, and de novo chimera detection conducted with UCHIME V.5.1.^43^ ^44^ Taxonomy will be assigned to OTUs using the naïve Bayesian RDP (Ribosomal Database Project) Classifier using the SILVA and GREENGENES taxonomic databases.

Estimates of within-sample species richness (number of OTUs) and diversity (Shannon index) at multiple rarefaction depths will be compared between infants who seroconvert and those who fail to seroconvert using Student's t-test. Weighted and unweighted UniFrac distances will be used to assess potential clustering of microbiota composition according to seroconversion status, as well as the degree of overlap in composition according to the age and geographical location. These results will be visualised using principal coordinates analyses and statistically significant clusters will be identified using Adonis. Random forests will be used to identify OTUs that distinguish individuals according to RV vaccine response, age and geographical location.

#### Effect of OPV coadministration on RV immunogenicity

The effect of administration of OPV on RV vaccine immunogenicity will be assessed by comparing seroconversion and geometric mean concentrations (GMC of R IgA) between infants in India in the OPV and IPV cohorts using the χ^2^ test and Student's t-test (or non-parametric equivalent), respectively.

## Discussion

Maternal antibodies to RV are higher in low-income countries and in both in trials of withholding breast feeding in India and elsewhere and animal models have demonstrated that maternal antibodies interfere to some extent with RV vaccine immune response.^14^ ^45^ ^46^ However, their comparative effect on the immunogenicity of RV vaccination among populations of the developing and developed world is currently unknown. Additionally, the relationship between systemic antibodies and secretory antibodies in breast milk is not known; although the studies of withholding breast feeding immediately prior to RV immunisation did not significantly impact the vaccine immunogenicity.^46^ ^47^ Levels of transplacental transfer of IgG to the infant may be influenced by risk factors such as the high prevalence of HIV in resource-poor settings irrespective of the high maternal RV-specific antibody levels from reinfections, such as may be understood from Malawi, with a maternal HIV prevalence of 20%.^48^ Despite efforts to study the interactions between commensal bacteria and host immune responses, the role of the intestinal microbiota on vaccine response remains underinvestigated, especially in heterogeneous populations. Evaluation of immunogenicity of oral RV vaccines given with OPV and potential for interference have been described from Bangladesh, but not with vaccination on a routine immunisation schedule.^49^ This study will evaluate performance of the RV vaccine in the routine schedule in two developing counties where OPV may be soon phased out to be replaced with IPV as per the Global Polio Eradication Initiative (GPEI) Strategic Plan for 2013–2018.

This study will provide information on multiple factors that may help to understand the performance of RV vaccines and thus develop strategies for improvement, especially where the vaccines are less effective in reducing the burden of RV gastroenteritis.

## Dissemination

Participant recruitment began in 2016 and follow-up of the mother–infant pairs is ongoing at all three sites. Analysis of data, followed by publication of the results, is expected in 2018.
